# Role of Tryptophan Metabolism in Cancer

**DOI:** 10.1002/cai2.70037

**Published:** 2025-11-30

**Authors:** Zhehao Cui, Dandan Wang, Ye Zhang, Long Yuan, Yi Zhang, Xiaowei Qi

**Affiliations:** ^1^ Department of Breast and Thyroid Surgery, Southwest Hospital Army Medical University Chongqing China; ^2^ Key Laboratory of Chongqing Health Commission for Minimally Invasive and Precise Diagnosis and Treatment of Breast Cancer Chongqing China

**Keywords:** angiogenesis, cancer treatment, drug resistance, immune evasion, tryptophan metabolism, tumorigenesis

## Abstract

Tryptophan (Trp) is an essential amino acid that serves as a precursor for the synthesis of several important bioactive compounds. Trp is involved in a variety of pathophysiological processes, including neuronal function, metabolism, inflammatory responses, oxidative stress, immune regulation, and intestinal homeostasis. The role of Trp metabolism in tumorigenesis and cancer progression is of particular significance. The influence of Trp and its metabolites on tumor growth and metastasis is mediated through various mechanisms, such as immune evasion, promotion of angiogenesis, and increased resistance to therapeutic agents. This review presents the physiological pathways involved in Trp metabolism and its implications for various malignancies. We also highlight the latest clinical research targeting Trp metabolic pathways in oncology, in addition to exploring future directions for therapeutic advancements aimed at modulating Trp metabolism to enhance cancer treatment outcomes.

## Graphical abstract

This review systematically summarizes the functions and related mechanisms of tryptophan metabolism in various tumor types. It presents the latest progress in this field in a comprehensive manner and combines this with clinical practice to offer a new perspective on tumor treatment.

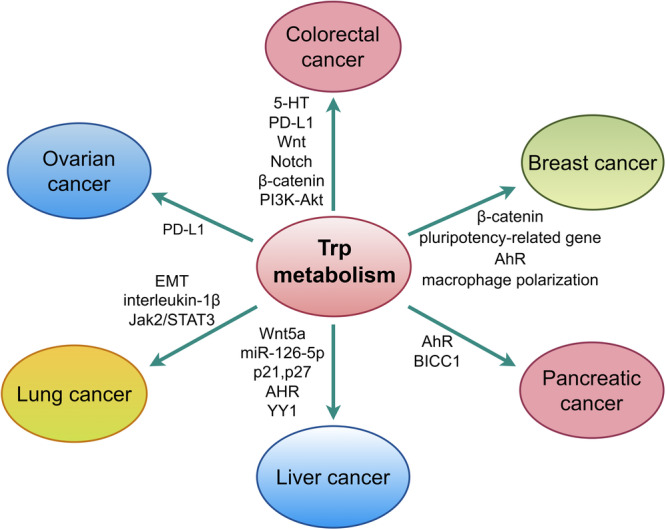

**Abbreviations**
5‐HT: 5‐hydroxytryptamine
AhR: aryl hydrocarbon receptor
EMT: epithelial–mesenchymal transition
GCN‐2: general control nonderepressible 2
HCC: hepatocellular carcinoma
IAA: indole‐3‐acetic acid
IDO: indoleamine 2,3‐dioxygenase
IFN: interferon
KMO: kynurenine 3‐monooxygenase
Kyn: kynurenine
PD‐L1: programmed death‐ligand 1
TDO: tryptophan 2,3‐dioxygenase
TNBC: triple‐negative breast cancer
TPH: tryptophan hydroxylase
Trp: tryptophan

## 1 Introduction

Tryptophan (Trp) plays a vital role in numerous physiological processes in humans [1, 2, 3]. It serves as a key precursor for the biosynthesis of 5‐hydroxytryptophan, a neurotransmitter, and contributes to the production of growth hormones in plants [4]. Furthermore, Trp functions as a natural sedative, regulating circadian rhythms and enhancing sleep quality [1, 5]. In animals, it aids plasma protein renewal, riboflavin activity, and niacin and hemoglobin synthesis. It also supports fetal antibody production during pregnancy, as well as lactation during breastfeeding.

**Figure 1:**
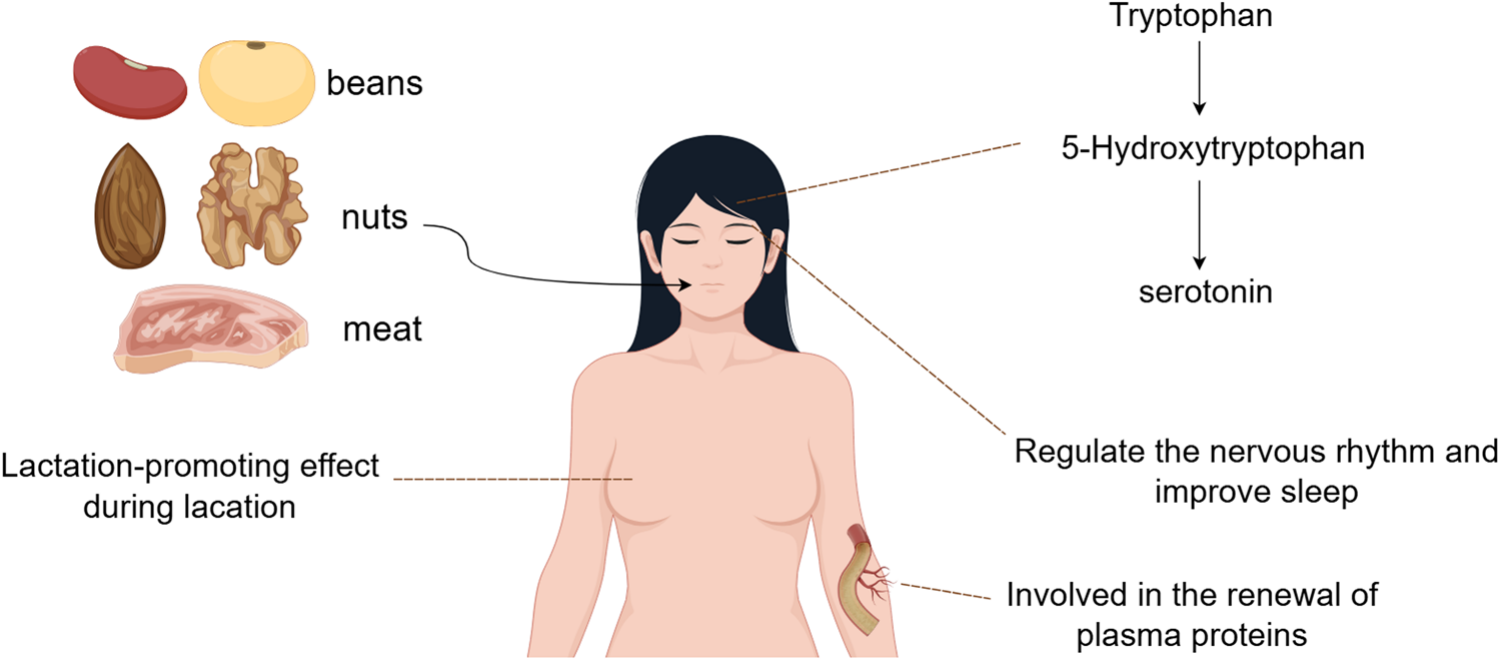
Natural sources of tryptophan and its physiological effects. By Figdraw.

Trp is abundant in legumes, nuts, and protein‐rich meats (e.g., chicken and beef), and adequate intake is necessary for optimal physiological functioning (Figure 1). Although amino acids primarily serve as substrates for protein synthesis, only a small fraction of Trp is utilized for this purpose. The majority is metabolized through diverse pathways to produce bioactive intermediates, which are involved in critical pathophysiological processes [1].

**Figure 2:**
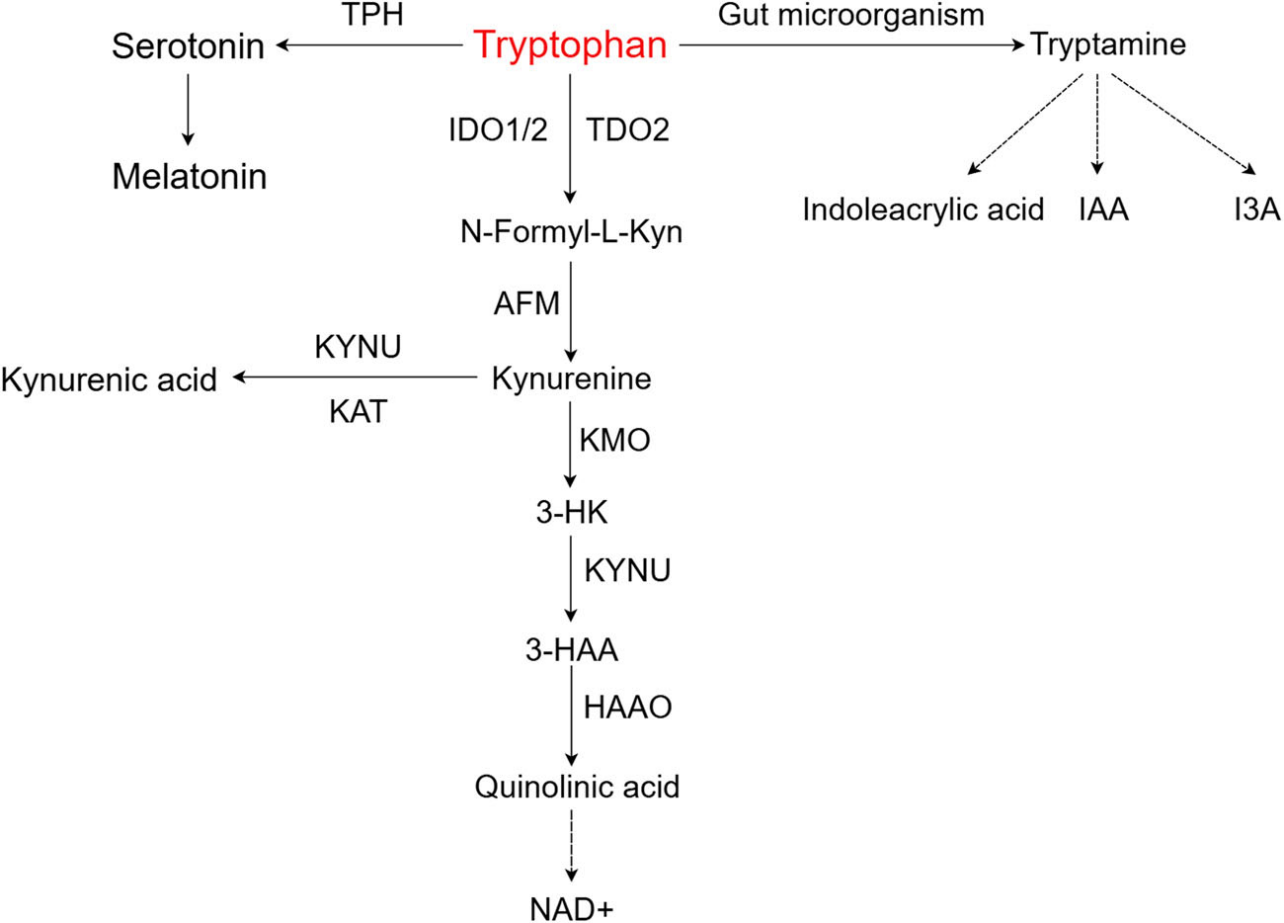
Tryptophan metabolic pathways. By Figdraw.

Trp metabolism proceeds through three major pathways (Figure 2):

- Kynurenine (Kyn) pathway: It is a predominant pathway, critical for immunomodulation, neurodevelopment, and brain function [6, 7]. Key rate‐limiting enzymes, including indoleamine 2,3‐dioxygenase (IDO), tryptophan 2,3‐dioxygenase (TDO), and kynurenine 3‐monooxygenase (KMO), regulate this pathway [8]. Major metabolites produced via this pathway include 3‐hydroxykynurenine, 3‐hydroxyanthranilic acid, N‐formylkynurenine, xanthurenic acid, kynurenic acid, anthranilic acid, and quinolinic acid.

- 5‐Hydroxytryptamine (5‐HT) pathway: This pathway involves key components, such as the 5‐HT transporter, 5‐HT receptor, tryptophan hydroxylase (TPH), and monoamine oxidase, which are essential for Trp hydroxylation. Principal products include 5‐hydroxyindoleacetic acid and 5‐HT. 5‐HT plays significant roles in regulating gastrointestinal motility, cardiovascular functions (e.g., vasoconstriction), and immune cell activity [9].

- Indole pathway: A critical component of Trp decarboxylation, this pathway is catalyzed by gut microorganisms and converts Trp into tryptamine, which is subsequently metabolized into indoleacetic acid derivatives, including indoleacrylic acid, indole‐3‐acetic acid (IAA), and indole‐3‐acetaldehyde. Indoles and their derivatives serve as natural ligands for the aryl hydrocarbon receptor (AhR), which is activated upon ligand binding. AhR activation regulates the transcription of downstream genes, with AhR–ligand complexes playing a pivotal role in various biological processes, including detoxification metabolism, immunomodulation, and developmental pathways. In addition, AhR regulates immune cell differentiation and functions, contributing to immune response regulation.

Trp is not only essential for maintaining normal physiological functions in humans but also implicated in the onset and progression of various diseases. This review provides a comprehensive overview of the roles and mechanisms of Trp metabolism in human health and diseases, with a particular emphasis on its involvement in malignant tumors. We also highlight recent clinical advancements in Trp‐targeted therapies and explore potential avenues for future research and therapeutic development in this field.

## 2 Trp Metabolism and Malignant Tumors

Trp metabolism is influenced by various factors, including genetic variations, diet, stress, exercise, and aging. These factors alter Trp metabolic pathways, thereby contributing to the progression of numerous diseases, including cancer. The role of Trp metabolism in tumorigenesis and cancer progression is widely reported.

**Figure 3:**
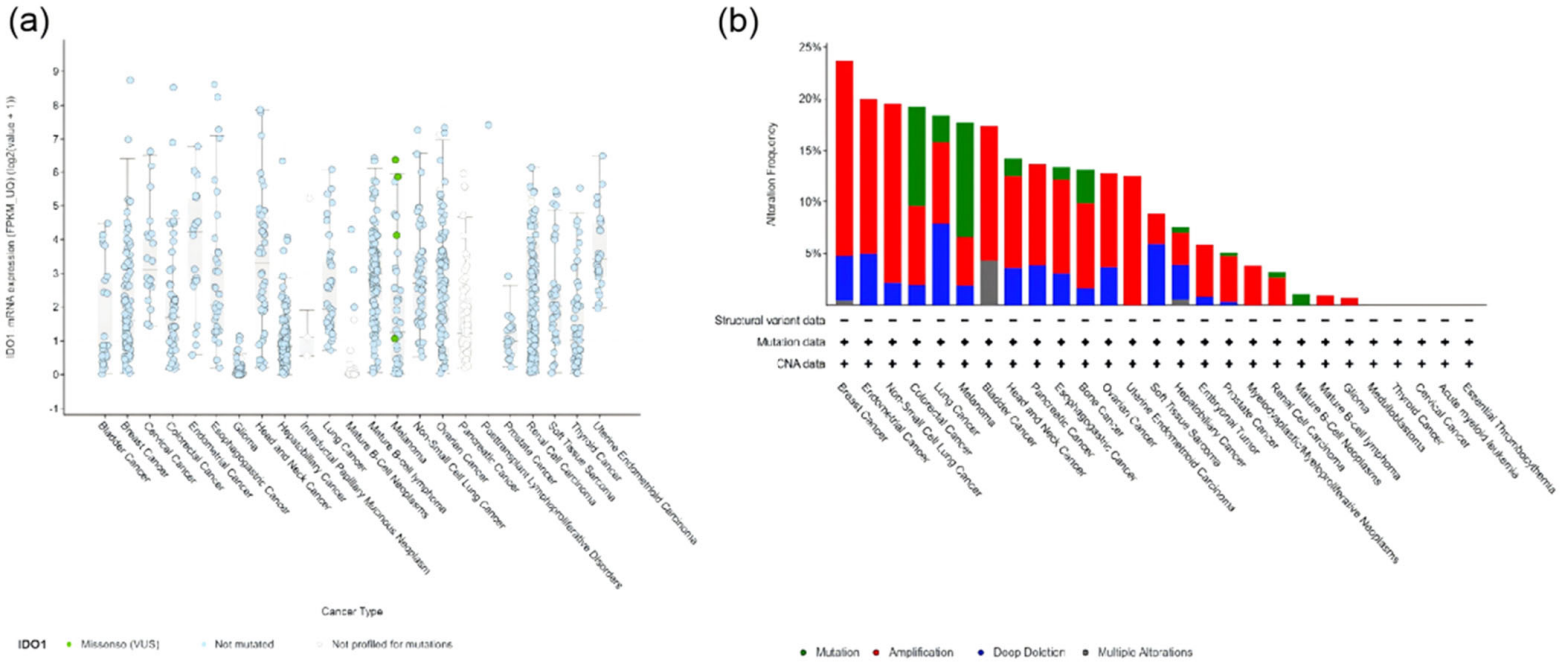
Manifestation of restriction enzymes in the Kyn pathway in different tumors: (a) IDO1 expression across various cancers. (b) Types and frequencies of IDO1/TDO2 mutations in different tumors.

Aberrant expression levels of Trp‐metabolizing enzymes are prevalent in the majority of cancers and are correlated with unfavorable disease outcomes. IDO1 and TDO2, essential enzymes in the Kyn metabolic pathway, are commonly overexpressed in numerous tumor types (Figure 3), including those affecting the pancreas, breast, and brain [10, 11, 12, 13]. Clinical studies highlight that the dysregulated expression of IDO1 and TDO2 significantly influences cancer prognosis. Raised IDO1 levels have been implicated in reduced survival rates in cancers, such as uterine corpus endometrial carcinoma. However, in specific cancer types like breast cancer, cervical squamous cell carcinoma, head and neck squamous cell carcinoma, ovarian cancer, lung adenocarcinoma, rectal adenocarcinoma, and sarcoma, elevated IDO1 expression is intriguingly associated with a more favorable prognosis (Supporting Information S1: Table 1). Similarly, heightened TDO2 expression has been associated with poor prognosis in cervical squamous cell carcinoma, kidney renal clear cell carcinoma, and testicular germ cell tumor. Interestingly, TDO2 overexpression has been linked to better outcomes in patients diagnosed with sarcoma (Supporting Information S1: Table 2).

The following sections detail the mechanisms through which Trp metabolism influences different types of malignant tumors.

### 2.1 Breast Cancer

**Figure 4:**
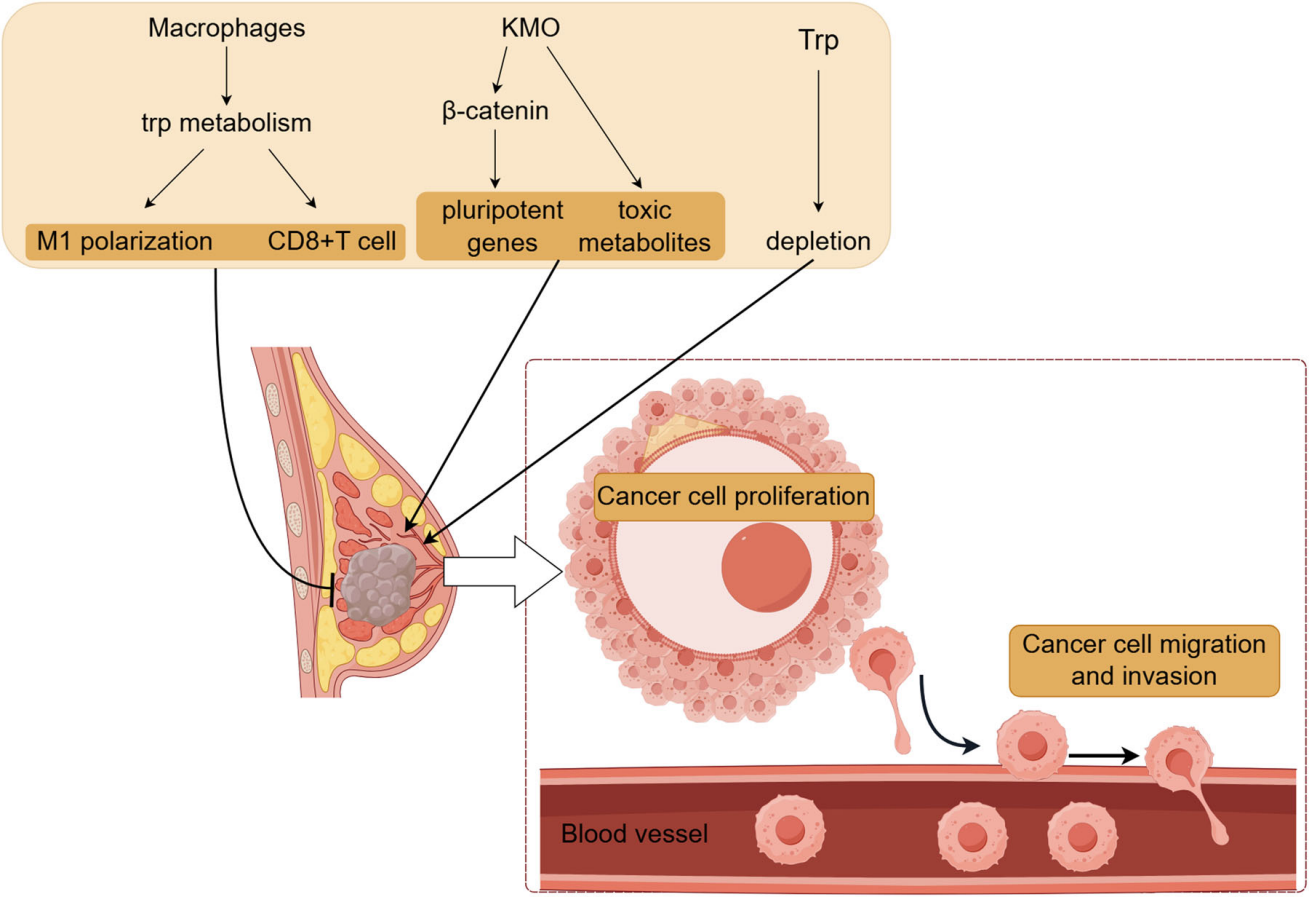
Molecular mechanisms underlying tryptophan metabolism and signaling in breast cancer. KMO, kynurenine 3‐monooxygenase. By Figdraw.

The Kyn pathway of Trp, a major Trp metabolic route, plays a pivotal role in breast cancer progression by promoting tumor immune evasion (Figure 4). Kyn induces CD8+ T‐cell apoptosis, as demonstrated by experiments using peripheral blood‐derived primary immune cells. Furthermore, elevated expression of IDO2 has been observed in breast cancer tissues compared with normal tissues. AhR‐mediated IDO2 expression seemingly contributes to a tumor‐promoting microenvironment in breast cancer [14, 15]. IDO1 expression has also been extensively studied in the context of breast cancer. While most studies associate high IDO1 expression with breast cancer progression [16, 17, 18, 19, 20, 21, 22, 23, 24, 25], conflicting evidence exists. For instance, one study reported an inverse correlation between IDO1 expression and cancer progression [26], while another found no significant difference in IDO1 expression between cancerous and noncancerous tissues [27]. Despite the potential of IDO1 as a target for tumor immunotherapy, clinical trials of IDO1 inhibitors are yet to show satisfactory outcomes [28].

The expression of KMO, another key enzyme in the Kyn pathway, is significantly upregulated in triple‐negative breast cancer (TNBC). KMO interacts with β‐catenin to prevent its degradation, promoting pluripotency‐related gene transcription and enhancing TNBC cell migration and invasion [29]. Inhibition of KMO reduces the production of toxic metabolites in the Kyn pathway and suppresses tumor growth, highlighting its potential as a therapeutic target, particularly for TNBC [30].

Adipocytes also contribute to Trp metabolism in breast cancer by metabolizing Trp and releasing Kyn, which activates the AhR in mammary epithelial cells, promoting malignant transformation. In vitro studies have reported that AhR inhibitors or AhR expression knockdown prevent this transformation [31].

Gut microbiota has emerged as a crucial factor in breast cancer development. Patients with breast cancer exhibit an abundance of *Prevotella*, particularly *Prevotella copri*, which exacerbates breast cancer progression by depleting Trp [32]. Furthermore, macrophage‐mediated Trp metabolism has been associated with M1 macrophage polarization, increasing CD8+ T‐cell populations and enhancing antitumor immune responses [33].

### 2.2 Hepatocellular Carcinoma (HCC)

In addition to breast cancer, Trp metabolism plays a crucial role in liver cancer, particularly HCC. High expression of TDO2 in HCC tissues promotes tumor cell migration and invasion through the Wnt5a signaling pathway [34]. TDO2‐mediated activation of the Kyn–AhR pathway facilitates epithelial–mesenchymal transition (EMT), driving HCC metastasis and invasion [35]. MicroRNAs and circular RNAs have been found to regulate TDO2 expression in HCC. For example, miR‐126‐5p overexpression enhances TDO2 expression, promoting tumor proliferation and metastasis [36]. Similarly, circZNF566 regulates TDO2 expression to drive HCC progression [37]. However, conflicting evidence exists: low TDO2 expression has been associated with poor prognosis in HCC, likely due to cell cycle arrest mediated by p21 and p27 upregulation [38]. IDO1 also contributes to HCC development by facilitating immune evasion through T‐cell modulation. IDO1 inhibition, using agents such as abrine, has demonstrated promise in preventing immune escape and enhancing antitumor efficacy in combination with anti‐programmed cell death protein‐1 (PD‐1) antibodies [39].

KMO expression is significantly elevated in HCC, correlating with poor prognosis, leading to its identification as a potential prognostic marker and therapeutic target [40]. Conversely, Trp side‐chain oxidase inhibits HCC growth by degrading Trp, offering an alternative therapeutic approach [41].

Several Trp metabolites exhibit distinct effects on HCC progression. For instance, 3‐hydroxyanthranilic acid, a Kyn pathway product, inhibits tumor growth by inducing apoptosis in HCC cells through interaction with the transcription factor YY1 [42]. Conversely, indole‐3‐pyruvate promotes liver tumorigenesis. A recent study reported that MYC‐driven liver tumors exhibit significantly increased Trp uptake. A low‐Trp diet markedly reduced tumor growth, whereas indole‐3‐pyruvate supplementation restored growth in Trp‐deprived HCC cells. These findings suggest that targeting indole‐3‐pyruvate or employing Trp deprivation strategies could offer novel therapeutic approaches for MYC‐driven liver tumors [43].

Complex interactions between gut microbiota and HCC have also been reported. Gut dysbiosis promotes liver tumorigenesis by modulating Trp metabolism and upregulating sterol regulatory element‐binding protein 2 expression. Interestingly, while gut microbiota depletion promotes liver tumorigenesis, it does not influence tumor progression, underscoring the intricate role of gut flora in HCC development [44].

### 2.3 Ovarian Cancer

IDO expression is significantly elevated in ovarian tumor cells, and high IDO expression correlates with poorer prognosis in patients with ovarian cancer [45]. IDO1 expression promotes ovarian cancer progression through AhR activation, inducing PD‐1 expression in T cells [46]. The aberrant expression of IDO in ovarian cancer highlights its potential as a therapeutic target [47]. Dual inhibition of TDO2 and IDO1 has been shown to reduce programmed death‐ligand 1 (PD‐L1) expression, alleviate immunosuppression, and improve survival outcomes [48].

The IDO inhibitor 1‐methyltryptophan has two enantiomers with distinct effects. While L‐1‐methyltryptophan downregulates IDO expression and inhibits tumor immune evasion, D‐1‐methyltryptophan paradoxically upregulates IDO expression in tumor cells, promoting immune escape [49, 50, 51]. Most IDO1 inhibitors in clinical trials have not demonstrated expected antitumor effects, possibly due to the nonenzymatic mechanisms of IDO1. For instance, epacadostat reduces IDO1 enzymatic activity but stabilizes its apolipoprotein form, activating a signaling pathway that promotes tumor growth [52].

### 2.4 Lung Cancer

IDO expression is significantly higher in lung cancer tissues compared with normal tissues, with elevated IDO levels and increased Kyn‐to‐Trp (Kyn/Trp) ratio associated with heightened carcinogenesis risk and poorer prognosis [53, 54, 55]. The inflammatory cytokine interleukin‐1β upregulates IDO1 expression, enhancing the conversion of Trp to Kyn, which exacerbates immunosuppression and tumor progression [56]. Moreover, IDO1 downregulates the natural killer group 2D ligand via ADAM10, impairing natural killer cell function and facilitating non‐small cell lung cancer progression [57].

The Trp metabolite D‐Kyn induces EMT in lung cancer through AhR activation [58]. Furthermore, AhR activation maintains non‐small cell lung cancer stem cells via the Jak2/STAT3 signaling pathway [59]. IDO1 serves as a prognostic biomarker, effectively predicting metastatic risk and survival outcomes in lung cancer [60, 61, 62, 63]. Emerging evidence suggests that the nonenzymatic roles of IDO2 also influence tumor progression, as it minimally contributes to Trp metabolism in cancer cells [64]. In addition, Trp depletion is associated with fatigue and reduced quality of life in patients with lung cancer, which further emphasizes the clinical significance of Trp metabolism in this context [65].

Preclinical studies have demonstrated the therapeutic potential of targeting IDO and TDO pathways in lung cancer. Inhibiting IDO1 expression enhances CD8+ T‐cell infiltration, reactivates antitumor immunity, and delays tumor growth [66]. Moreover, TDO2 expression is associated with lung adenocarcinoma cell proliferation, survival, and invasion, suggesting it as a therapeutic target [67]. IDO1 inhibitors have shown greater efficacy in cisplatin‐resistant lung cancer patients [68]. The IDO1 inhibitor LY3381916 is undergoing phase I clinical trials in advanced cancers [69].

Emerging therapeutic strategies for lung cancer are also showing considerable promise. For instance, ginseng polysaccharides have been found to modulate gut microbiota and alter the Kyn/Trp ratio, enhancing the efficacy of anti‐PD‐1 immunotherapy in patients with lung cancer [70]. Another promising candidate is 5‐methoxytryptophan, a Trp metabolite that suppresses EMT, cancer cell migration, and metastasis by inhibiting cyclooxygenase‐2 expression [71]. Notably, combining sorafenib with 5‐methoxytryptophan has been reported to significantly enhance the inhibition of lung cancer cell migration and invasion compared with either treatment alone [72].

### 2.5 Colorectal Cancer (CRC)

Patients with CRC exhibit significantly elevated serum Kyn/Trp ratios, and higher ratios are associated with an elevated risk of severe complications [73, 74]. Plasma Trp levels inversely correlate with colon cancer risk, while 5‐HT levels positively correlate with increased risk [75]. Reduced serum Trp levels also impact patient quality of life [76, 77]. Elevated IDO1, TDO2, and KMO expression is reportedly correlated with CRC metastasis and poorer prognosis [78, 79].

The mechanisms via which enzymes, such as IDO1, TDO2, and Kyn, promote CRC progression are well characterized, with the most critical pathway involving the activation of the IDO/TDO2‐Kyn‐AhR axis. This activation mediates regulatory T‐cell (Treg) differentiation and promotes inflammation‐associated colon cancer development [80]. In addition, the TDO2‐Kyn‐AhR pathway facilitates hepatic metastasis of CRC through PD‐L1‐mediated immune escape [81]. Kyn promotes vesicle cell differentiation in HT‐29 colon cancer cells by modulating Wnt, Notch, and AhR signaling pathways [82]. Kyn and quinolinic acid activate β‐catenin, promoting colon cancer cell proliferation and tumor growth in mouse models [83]. Furthermore, IDO1 and Kyn pathway metabolites activate PI3K‐Akt signaling cascade in neoplastic colonic epithelial cells, promoting cancer cell proliferation and inhibiting apoptosis [84].

IDO1 and TDO2 have emerged as promising therapeutic targets in CRC. Elevated IDO1 activity reduces the radiosensitivity of CRC cells, potentially impairing radiotherapy efficacy. This highlights the potential of IDO1 inhibitors as adjuvant therapies to improve radiotherapy outcomes in patients with CRC [85]. Besides, IDO inhibitors have been found to augment the antitumor activity of Toll‐like receptor agonists [86].

Notably, IDO inhibitors, such as 1‐methyltryptophan and epigallocatechin gallate, have demonstrated efficacy in preventing the formation of colonic preneoplastic lesions, representing a novel strategy for colon cancer prevention [87]. The IDO inhibitor D‐1‐methyltryptophan enhances the antitumor efficacy of oxaliplatin [88], while the combination of a stimulator of interferon (IFN) genes with an IDO inhibitor significantly suppresses CRC progression [89]. Another IDO inhibitor, L‐1‐methyltryptophan, induces mitotic death in colon cancer cells by inhibiting the transcription of CDC20 [90]. Similarly, TDO2 knockdown reportedly inhibits CRC progression via the TDO2‐Kyn‐AhR pathway [91]. Moreover, TDO inhibitors have been shown to enhance the efficacy of immune checkpoint inhibitors, presenting a potential combinatory therapeutic strategy for CRC [92].

In addition to metabolic enzymes, other molecular targets hold therapeutic promise in CRC. The Trp metabolite 8‐hydroxyquinaldic acid has been shown to exert antiproliferative and antimigratory effects on CRC cells [93]. Similarly, 5‐methoxytryptophan promotes apoptosis, induces cell cycle arrest, and inhibits cell proliferation, suggesting its potential as a therapeutic agent for CRC [94]. Inhibition of 5‐HT uptake via blockade of the 5‐HT transporter has also demonstrated antitumor effects in CRC [95]. Furthermore, metformin, by reprogramming Trp metabolism, enhances the functional activity of CD8+ T cells, offering a potential immunotherapeutic approach for CRC treatment [96].

Gut microbiota plays a critical role in CRC progression by modulating Trp metabolism. For instance, indole‐3‐carboxaldehyde, a metabolite derived from *Lactobacillus gallinarum*, improves anti‐PD‐1 therapy efficacy in CRC by inhibiting the differentiation of CD4+ Tregs and modulating the IDO1/Kyn/AhR axis to enhance CD8+ T‐cell function [97]. Similarly, *Akkermansia muciniphila*, a Gram‐negative anaerobic bacterium, suppresses CRC progression by targeting the Trp‐mediated AhR/β‐catenin signaling pathway [98].

Statins, widely known for their cholesterol‐lowering effects, exhibit chemopreventive potential in CRC. Their efficacy is enhanced by the intestinal commensal bacterium *Lactobacillus reuteri* [99]. Moreover, microbiota‐mediated alterations in fecal Trp metabolism have been linked to intestinal barrier function and localized Trp metabolic processes, contributing to CRC pathogenesis [100].

### 2.6 Melanoma

Patients with melanoma exhibit significantly higher serum Kyn/Trp ratios compared with healthy individuals [101]. Elevated serum Kyn/Trp ratios, coupled with increased expression of IDO1, are associated with poor prognosis [102, 103]. IDO1, traditionally recognized for catalyzing Trp metabolism, influences tumor progression. A recent study suggested that IDO1 also acts as a signaling molecule, promoting malignant progression in melanoma, offering new therapeutic insights [104].

IDO, PD‐L1, and cytotoxic T‐lymphocyte‐associated protein 4 expression in the peripheral blood of melanoma patients is closely interconnected [105]. Notably, the upregulation of PD‐L1, IDO, and regulatory Tregs is driven by CD8+ T cells [106], suggesting that combination therapies targeting these markers may yield synergistic antitumor effects, particularly in tumor microenvironments with pre‐existing T‐cell inflammation.

As with other malignancies, research on Trp metabolism in melanoma has predominantly focused on IDO inhibitors. For instance, the combination of the IDO pathway inhibitor indoximod with pembrolizumab has shown antitumor efficacy in phase II clinical trials for advanced melanoma [107]. Similarly, the small‐molecule IDO inhibitor NTRC 3883‐0 was found to exhibit antitumor activity in a melanoma mouse model [108]. The combination of IDO inhibitors, such as L‐MT and NLG919, with pembrolizumab and paclitaxel, respectively, has resulted in enhanced antitumor effects, underscoring the potential of IDO inhibitor‐based combination therapies [109, 110]. Novel delivery systems, such as cationic liposomes co‐delivering tumor vaccines and IDO inhibitors, have further augmented antitumor T‐cell responses, highlighting their potential in combination immunotherapy [111].

Despite these advancements, many clinical trials have failed to meet expectations. For instance, the IDO inhibitor epacadostat was shown to inhibit both the enzymatic and nonenzymatic (signaling) functions of IDO1, which may partially explain its limited efficacy [112]. Another study indicated that IDO1 inhibition led to aberrant expression of IFN‐γ and microphthalmia‐associated transcription factor, dampening T‐cell‐mediated antitumor responses and promoting tumor survival [113].

**Figure 5:**
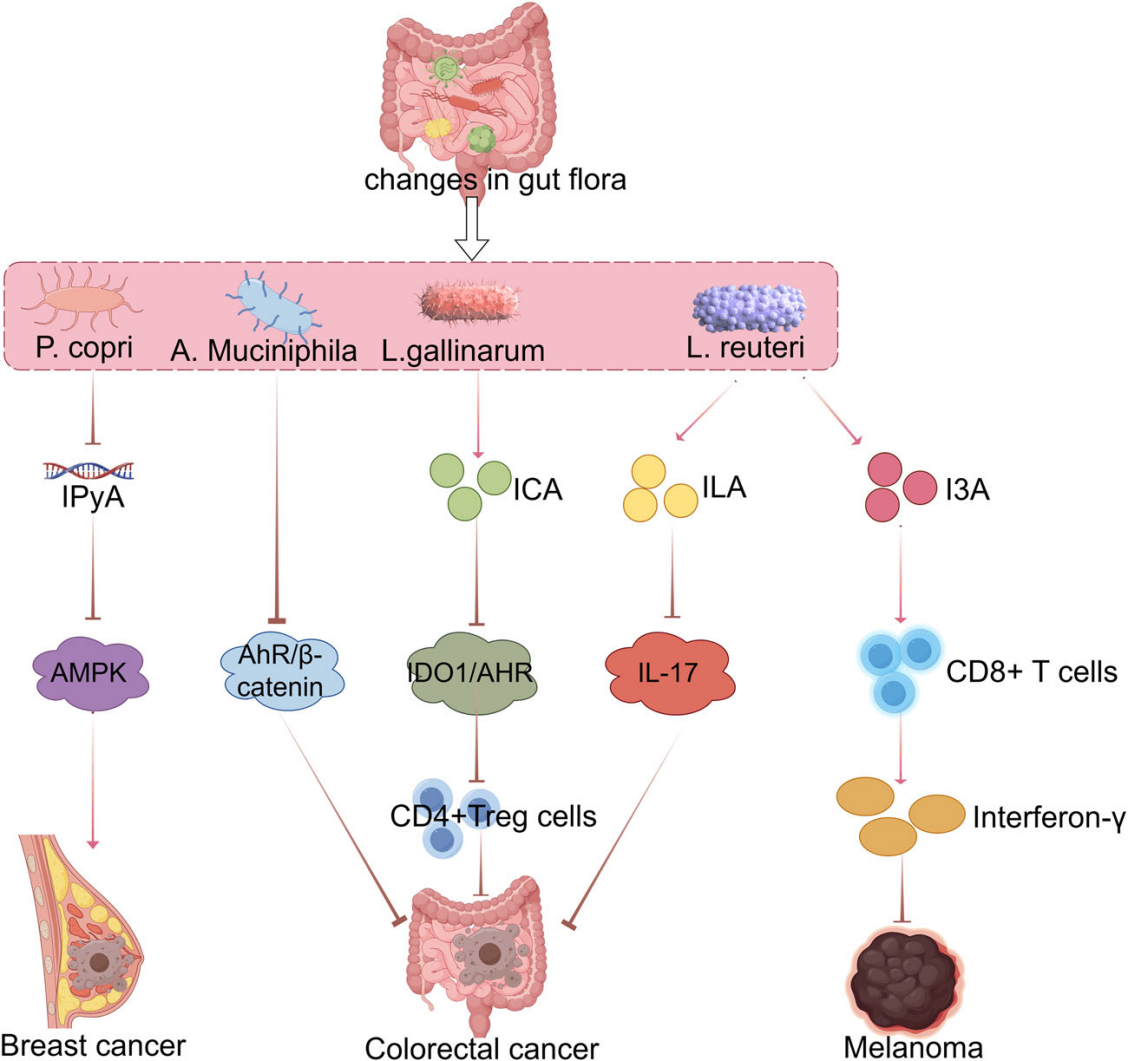
Gut microbiota influences tumor progression by affecting tryptophan metabolism. By Figdraw.

Gut microbiota also influences melanoma progression through metabolites such as indole‐3‐carboxaldehyde secreted by *Lactobacillus rohita* (Figure 5). Indole‐3‐carboxaldehyde activates the AhR signaling pathway in CD8+ T cells, enhancing IFN‐γ production and improving the efficacy of immune checkpoint inhibitors. Trp‐rich diets further enhance these antitumor effects, particularly in the presence of a diverse microbiota [114].

### 2.7 Pancreatic Cancer

Clinical studies reveal that plasma Trp levels are significantly lower in patients with pancreatic cancer compared with healthy individuals [115]. These reductions are linked to cancer cachexia, a condition characterized by severe weight loss and muscle wasting [116]. Conversely, Trp‐enriched diets have been associated with a reduced risk of pancreatic cancer [117, 118].

Microbiota‐derived metabolites, such as IAA, enhance chemotherapy efficacy and increase treatment sensitivity in chemotherapy‐resistant pancreatic ductal adenocarcinoma [119]. However, the effects of Trp metabolites on pancreatic cancer are complex. While metabolites such as IAA exhibit therapeutic potential, elevated Kyn levels correlate with reduced survival rates [120].

AhR expression is markedly elevated in patients with pancreatic cancer compared with healthy controls, and high AhR expression is associated with accelerated disease progression and enhanced immunosuppression [121]. In addition, Trp metabolism may contribute to depression in patients with pancreatic cancer, complicating disease management [122].

IDO1 overexpression is a significant immunomodulatory factor in pancreatic cancer, representing a primary therapeutic target [123]. For example, carbidopa, an AhR agonist, suppresses IDO1 expression in pancreatic cancer cells, attenuating tumor growth [124]. The RNA‐binding protein BICC1 enhances chemoresistance in pancreatic cancer by upregulating IDO1 expression, suggesting that targeting the BICC1/IDO1/Trp metabolic axis could help overcome drug resistance [125]. Furthermore, IDO1 inhibition has been shown to alleviate depressive symptoms in patients with pancreatic cancer [126].

### 2.8 Other Tumors

TDO2 overexpression has been documented in gliomas, where it drives Trp metabolism to Kyn, activating the AhR and downstream PI3K/AKT signaling pathways to promote tumor stemness and growth [127]. AhR activation also facilitates EMT in gliomas through a transforming growth factor‐β‐dependent mechanism, increasing tumor invasiveness [127].

In multiple myeloma, targeting KMO enhances antitumor immunity and cytotoxicity [128]. In renal cell carcinoma, the polyadenylate‐binding protein PABPC1L facilitates immune evasion by upregulating IDO1 expression, impairing T‐cell function, and presenting a potential target for immune checkpoint blockade therapies [129]. TDO2 promotes tumor progression in bladder cancer via AhR‐mediated SPARC/FILIP1L signaling [130]. In gastric cancer, glutathione peroxidase‐2 supports tumor progression through the KYNU‐Kyn‐AhR signaling pathway, while glutathione peroxidase‐2 knockdown suppresses tumor growth and metastasis [131]. Elevated TDO2 expression in prostate cancer drives Trp metabolism and Kyn production, activating the AhR/c‐Myc/ABC signaling pathway and contributing to drug resistance. Disrupting this pathway has been shown to mitigate TDO2‐induced drug resistance [132].

A recent study proposed combining lactate depletion‐induced starvation therapy, photothermal therapy, and IDO inhibition to suppress tumor growth, reverse immunosuppression, and prevent tumor metastasis [133].

## 3 Trp Metabolism in Immune Regulation and Tumor Progression

T‐lymphocytes are essential components of the adaptive immune system, categorized into distinct subpopulations based on their functions. They are broadly classified into CD4+ helper T cells, which assist in immune regulation, and CD8+ cytotoxic T cells, primarily recognized for their antitumor activity. The AhR is a ligand‐activated transcription factor triggered by various Trp‐derived metabolites. Once activated, the AhR plays a pivotal role in immune regulation and numerous cellular processes. Metabolites from the Kyn pathway modulate immune responses by activating the AhR signaling pathway, influencing the differentiation, population dynamics, and functional status of T‐lymphocytes, ultimately impacting tumor development.

**Figure 6:**
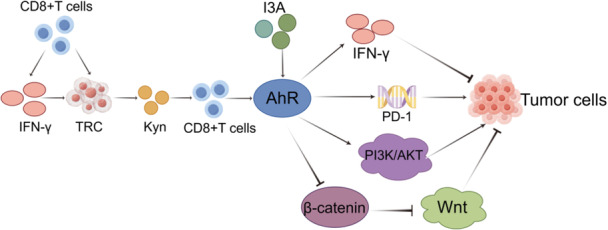
Tryptophan metabolism exerts immunomodulatory effects by activating the AhR pathway, influencing tumor progression. By Figdraw.

The activation of the AhR pathway is associated with CD8+ T‐cell exhaustion, suppression of antitumor immune responses, facilitation of immune escape, and promotion of tumor progression. Tumor‐reproducing cells drive PD‐1 upregulation in CD8+ T cells via the transcellular Kyn‐AhR pathway, amplifying both immunosuppressive and protumor effects [134]. In contrast, some studies highlight the tumor‐suppressive potential of AhR pathway activation. This is primarily mediated by the proteasomal degradation of β‐catenin, which limits Wnt pathway activity [98, 135, 136]. In addition, recent findings underscore the role of indole‐3‐carboxaldehyde in activating the AhR pathway in CD8+ T cells. This activation enhances the production of IFN‐γ, a cytokine critical for cancer cell cytotoxicity and for improving the efficacy of immune checkpoint inhibitors (Figure 6) [114].

In addition to the AhR pathway, the depletion of Trp in the tumor microenvironment can also exert an immunomodulatory effect. Research has demonstrated that IDO1‐mediated Trp depletion in dendritic cells can lead to significant anergy in CD8+ T cells and the transformation of immature CD4+ T cells into Treg cells via the amino acid starvation sensor general control nonderepressible 2 (GCN2) kinase [137]. GCN2 is critical for the polarization of immunosuppressive TAMs and myeloid‐derived suppressor cells (MDSCs). Moreover, the activation of GCN2 by IDO1 is essential for the secretion of IL‐10 and TGF‐β by splenic macrophages and the establishment of peripheral tolerance to apoptotic cells [138].

Another study indicated that IDO expression recruits immunosuppressive regulatory T cells (Tregs) and simultaneously reduces the frequency of CD8+ T cells. This immune imbalance triggers immunosuppression and tumor growth. Furthermore, elevated IDO expression results in increased Trp consumption and Kyn accumulation, creating a local immunosuppressive environment that inactivates T cells and facilitates the proliferation of glioma cells [139].

## 4 Current and Future Clinical Development of IDO, TDO and Other Inhibitors

As the role of Trp metabolism in tumor development becomes increasingly well‐defined, clinical efforts targeting key enzymes, such as IDO and TDO, have intensified. IDO1 inhibitors, in particular, have undergone extensive evaluation in clinical trials, demonstrating potential for enhancing the efficacy of existing cancer therapies. Despite promising preclinical results, most late‐stage clinical trials have produced less favorable outcomes, highlighting the need for a deeper understanding of Trp metabolism and its multifaceted role in cancer progression.

### 4.1 Current Status of IDO and TDO Inhibitors

Trp metabolism is regulated by three main rate‐limiting enzymes: IDO1, IDO2, and TDO. Elevated IDO1 expression, a common feature in a broad spectrum of tumors, is often associated with poorer prognoses, making IDO1 a valuable therapeutic target. By suppressing antitumor immune responses, high IDO1 expression facilitates tumor progression, indicating that combining IDO1 inhibitors with immunotherapy may offer a promising strategy for cancer treatment.

Preclinical studies on IDO1 inhibitors have reported encouraging results, with several inhibitors advancing to the clinical trial stage (Supporting Information S1: Table 3). These include epacadostat [140, 141, 142, 143, 144, 145, 146, 147, 148, 149, 150, 151, 152, 153], indoximod [147, 154, 155, 156], BMS‐986205, PF‐06840003, navoximod [157, 158], and KHK2455. However, despite early promise, many clinical trials have failed to meet expectations [147, 159, 160]. As of now, no inhibitor has been identified that specifically targets TDO. Recent advancements in research have led to the exploration of dual inhibitors targeting both IDO1 and TDO. However, this study domain is still in its nascent phase. HTI‐1090 stands out as a novel, highly potent dual inhibitor effective against both IDO1 and TDO. It demonstrated promising safety profiles and oral bioavailability in preclinical trials. A Phase I multicenter clinical trial (NCT03208959) is presently ongoing to ascertain the preliminary efficacy and safety of HTI‐1090 in patients with advanced solid tumors [161].

### 4.2 Current Status of Other Inhibitors

Besides IDO and TDO, KMO and TPH are essential enzymes in the metabolism of Trp. Given that KMO is overexpressed in various cancer types and contributes to cancer progression, the creation of KMO inhibitors emerges as an innovative approach for cancer therapy. Nevertheless, the majority of KMO inhibitors currently being studied are aimed at neurodegenerative conditions, with no reported clinical trials specifically for cancer treatment. Likewise, TPH inhibitors have demonstrated potential efficacy in addressing neuropsychiatric disorders, gastrointestinal issues, osteoporosis, and additional medical conditions [162].

### 4.3 Future Perspectives

The limited success of IDO1 inhibitors in clinical trials may be attributed to several factors, including insufficient pharmacological inhibition of IDO1 and incomplete blockade of its downstream pathways. Future research should prioritize the development of drugs with enhanced pharmacological potency, patient stratification based on IDO1 expression levels, and simultaneous targeting of co‐dependent molecular pathways. Moreover, the nonenzymatic immunomodulatory activity of IDO1 might contribute to the suboptimal efficacy of current inhibitors. Further in vivo studies are warranted to elucidate the precise mechanisms underlying IDO1‐mediated immunomodulation. Combination inhibitors of IDO1 and TDO may broadly suppress Trp metabolism, potentially mitigating the limitations associated with selective IDO1 inhibition. Therefore, clinical trials focusing on dual inhibitors of IDO1 and TDO could represent a future direction. Studies on KMO and TPH inhibitors are still in the initial stages. Moving forward, advancing research on KMO and TPH inhibitors in cancer therapy is essential, offering a fresh viewpoint on targeted Trp metabolism treatment for cancer.

## 5 Summary

Trp plays a crucial role in various physiological processes. In cancer, Trp and its metabolites influence tumor progression by exerting immunomodulatory effects through diverse pathways. Emerging evidence highlights the role of gut microbiota in Trp metabolism, suggesting this axis as a potential therapeutic target in oncology.

Trp metabolism is mediated by three major pathways, with enzymes such as IDO1, IDO2, TDO2, and KMO serving as key regulators. Aberrant expression of these enzymes has been observed in various cancers, resulting in dysregulated Trp metabolism. Elevated enzyme expression often correlates with poor prognosis, making these enzymes attractive targets for therapeutic intervention.

Research on inhibitors of these enzymes has shown promise in preclinical and early clinical trials. However, less favorable outcomes in recent late‐stage clinical trials highlight significant challenges that remain unresolved. As inhibitors of Trp‐metabolizing enzymes can modulate tumor immunity, their combination with immunotherapy has emerged as a promising strategy. Such combination therapies represent a promising avenue for future cancer treatment development.

## Author Contributions

**Zhehao Cui:** visualization (equal), writing – original draft (equal), writing – review and editing (equal). **Dandan Wang:** methodology (equal), writing – original draft (equal). **Ye Zhang:** resources (equal), visualization (equal). **Long Yuan:** formal analysis (equal), visualization (equal). **Yi Zhang:** conceptualization (equal), methodology (equal), writing – review and editing (equal). **Xiaowei Qi:** methodology (equal), resources (equal), visualization (equal), writing – review and editing (equal).

## Ethics Statement

The authors have nothing to report.

## Consent

The authors have nothing to report.

## Conflicts of Interest

The authors declare no conflicts of interest.

## Supporting information


**Supplemental Table 1:** Correlation between IDO1 and overall survival in patients with cancer. **Supplemental Table 2:** Correlation between TDO2 and overall survival in patients with cancer. **Supplemental Table 3:** Mechanisms of action and clinical trial‐related data for various IDO1 inhibitors.

## Data Availability

Data sharing is not applicable to this article, as no data sets were generated or analyzed during the current study.
