# Supplementary material for: Role of Tryptophan Metabolism in Cancer
Source: Cancer Innov. 2025 Nov 30;4(6):e70037. doi: 10.1002/cai2.70037 (PMC12665871; doi:10.1002/cai2.70037)
Supplement: Supplementary file 1 — Supplemental Table 1: Correlation between IDO1 and overall survival in patients with cancer. Supplemental Table 2: Correlation between TDO2 and overall survival in patients with cancer. Supplemental Table 3: Mechanisms of action and clinical trial‐related data for various IDO1 inhibitors. [file CAI2-4-e70037-s001.docx]

**SUPPLEMENTAL TABLE 1** Correlation between IDO1 and overall survival in patients with cancer

| **Symbol** | **Cancer type** | **Prognosis** | **Endpoint** | ***p* value** | **Case** | **Dataset** | **Method** |
| --- | --- | --- | --- | --- | --- | --- | --- |
| IDO1 | Bladder carcinoma | – | Overall survival | 0.057 | 404 | TCGA | RNA-seq |
| IDO1 | Breast cancer | Good | Overall survival | 0.027 | 1089 | TCGA | RNA-seq |
| IDO1 | Cervical squamous cell carcinoma | Good | Overall survival | 0.017 | 304 | TCGA | RNA-seq |
| IDO1 | Esophageal adenocarcinoma | – | Overall survival | 0.13 | 80 | TCGA | RNA-seq |
| IDO1 | Esophageal squamous cell carcinoma | – | Overall survival | 0.076 | 81 | TCGA | RNA-seq |
| IDO1 | Head-neck squamous cell carcinoma | Good | Overall survival | 0.011 | 499 | TCGA | RNA-seq |
| IDO1 | Kidney renal clear cell carcinoma | – | Overall survival | 0.13 | 530 | TCGA | RNA-seq |
| IDO1 | Kidney renal papillary cell carcinoma | – | Overall survival | 6.6e-7 | 287 | TCGA | RNA-seq |
| IDO1 | Liver hepatocellular carcinoma | – | Overall survival | 0.23 | 370 | TCGA | RNA-seq |
| IDO1 | Lung adenocarcinoma | Good | Overall survival | 0.047 | 504 | TCGA | RNA-seq |
| IDO1 | Lung squamous cell carcinoma | – | Overall survival | 0.21 | 495 | TCGA | RNA-seq |
| IDO1 | Ovarian cancer | Good | Overall survival | 0.00033 | 373 | TCGA | RNA-seq |
| IDO1 | Pancreatic ductal adenocarcinoma | – | Overall survival | 0.089 | 177 | TCGA | RNA-seq |
| IDO1 | Pheochromocytoma and paraganglioma | – | Overall survival | 0.2 | 178 | TCGA | RNA-seq |
| IDO1 | Rectum adenocarcinoma | Good | Overall survival | 0.0079 | 165 | TCGA | RNA-seq |
| IDO1 | Sarcoma | Good | Overall survival | 0.00014 | 259 | TCGA | RNA-seq |
| IDO1 | Stomach adenocarcinoma | – | Overall survival | 0.054 | 371 | TCGA | RNA-seq |
| IDO1 | Testicular Germ Cell Tumor | – | Overall survival | 0.11 | 134 | TCGA | RNA-seq |
| IDO1 | Thymoma | – | Overall survival | 0.076 | 118 | TCGA | RNA-seq |
| IDO1 | Thyroid carcinoma | – | Overall survival | 0.12 | 502 | TCGA | RNA-seq |
| IDO1 | Uterine corpus endometrial carcinoma | Poor | Overall survival | 0.009 | 542 | TCGA | RNA-seq |

Source: https://kmplot.com/

**SUPPLEMENTAL TABLE 2** Correlation between TDO2 and overall survival in patients with cancer

| **Symbol** | **Cancer type** | **Prognosis** | **Endpoint** | ***p* value** | **Case** | **Dataset** | **Method** |
| --- | --- | --- | --- | --- | --- | --- | --- |
| TDO2 | Bladder carcinoma | – | Overall survival | 0.063 | 404 | TCGA | RNA-seq |
| TDO2 | Breast cancer | – | Overall survival | 0.08 | 1089 | TCGA | RNA-seq |
| TDO2 | Cervical squamous cell carcinoma | Poor | Overall survival | 0.0076 | 304 | TCGA | RNA-seq |
| TDO2 | Esophageal adenocarcinoma | – | Overall survival | 0.3 | 80 | TCGA | RNA-seq |
| TDO2 | Esophageal squamous cell carcinoma | – | Overall survival | 0.19 | 81 | TCGA | RNA-seq |
| TDO2 | Head-neck squamous cell carcinoma | – | Overall survival | 0.35 | 499 | TCGA | RNA-seq |
| TDO2 | Kidney renal clear cell carcinoma | Poor | Overall survival | 0.00038 | 530 | TCGA | RNA-seq |
| TDO2 | Kidney renal papillary cell carcinoma | – | Overall survival | 8.2e-7 | 287 | TCGA | RNA-seq |
| TDO2 | Liver hepatocellular carcinoma | – | Overall survival | 0.12 | 370 | TCGA | RNA-seq |
| TDO2 | Lung adenocarcinoma | – | Overall survival | 0.21 | 504 | TCGA | RNA-seq |
| TDO2 | Lung squamous cell carcinoma | – | Overall survival | 0.21 | 495 | TCGA | RNA-seq |
| TDO2 | Ovarian cancer | – | Overall survival | 0.099 | 373 | TCGA | RNA-seq |
| TDO2 | Pancreatic ductal adenocarcinoma | – | Overall survival | 0.35 | 177 | TCGA | RNA-seq |
| TDO2 | Pheochromocytoma and paraganglioma | – | Overall survival | 0.11 | 178 | TCGA | RNA-seq |
| TDO2 | Rectum adenocarcinoma | – | Overall survival | 0.24 | 165 | TCGA | RNA-seq |
| TDO2 | Sarcoma | Good | Overall survival | 0.0057 | 259 | TCGA | RNA-seq |
| TDO2 | Stomach adenocarcinoma | – | Overall survival | 0.075 | 371 | TCGA | RNA-seq |
| TDO2 | Testicular Germ Cell Tumor | Poor | Overall survival | 0.027 | 134 | TCGA | RNA-seq |
| TDO2 | Thymoma | – | Overall survival | 0.097 | 118 | TCGA | RNA-seq |
| TDO2 | Thyroid carcinoma | – | Overall survival | 0.13 | 502 | TCGA | RNA-seq |
| TDO2 | Uterine corpus endometrial carcinoma | – | Overall survival | 0.52 | 542 | TCGA | RNA-seq |

Source: https://kmplot.com/

**SUPPLEMENTAL TABLE 3** Mechanisms of action and clinical trial-related data for various IDO1 inhibitors

| **Drug** | **Mechanism** | **Cancer type** | **Phase** | **Identifier** | **References** |
| --- | --- | --- | --- | --- | --- |
| Epacadostat | Competitive inhibition of IDO1 | NSCLC | Ⅰ | NCT03322540 | [140] |
|  |  | ST | Ⅰ | NCT02559492 | [141] |
|  |  | ST | Ⅰ/Ⅱ | NCT02178722 | [142] |
|  |  | ST | Ⅰ/Ⅱ | NCT02318277 | [143] |
|  |  | ST | Ⅰ/Ⅱ | NCT02959437 | [144] |
|  |  | MM | Ⅰ/Ⅱ | NCT01604889 | [145] |
|  |  | UC | Ⅰ/Ⅱ | NCT03361865 | [146] |
|  |  | BC | Ⅱ | NCT01792050 | [147] |
|  |  | SAR | Ⅱ | NCT03414229 | [148] |
|  |  | OCCC | Ⅱ | NCT03602586 | [149] |
|  |  | NSCLC | Ⅱ | NCT03322566 | [150] |
|  |  | HNSCC | Ⅲ | NCT03358472 | [151] |
|  |  | UC | Ⅲ | NCT03374488 | [152] |
|  |  | MM | Ⅲ | NCT02752074 | [153] |
|  |  | MRCC | Ⅲ | NCT03260894 | [154] |
| Indoximod | Modulation of effector signaling  molecules of the IDO1 pathway | DIPG | Ⅰ | NCT02502708 | [155] |
|  |  | MT | Ⅰ | NCT00567931 | [156] |
|  |  | BC | Ⅱ | NCT01792050 | [147] |
|  |  | BC | Ⅰ/Ⅱ | NCT01042535 | [157] |
| Navoximod | Noncompetitive inhibition of IDO1 | ST | Ⅰ | NCT02471846 | [158] |
|  |  | ST | Ⅰ | NCT02048709 | [159] |

Note: NSCLC: non-small cell lung cancer, ST: solid tumor, MM: malignant melanoma, UC: urothelial carcinoma, BC: breast cancer, SAR: sarcoma, OCCC: ovarian clear cell carcinoma, HNSCC: head and neck squamous cell carcinoma, MRCC: metastatic renal cell carcinoma, DIPG: diffuse intrinsic pontine glioma, MT: mesenchymal tumor
